# The journals of importance to UK clinicians: a questionnaire survey of surgeons

**DOI:** 10.1186/1472-6947-6-24

**Published:** 2006-06-08

**Authors:** Teresa H Jones, Steve Hanney, Martin J Buxton

**Affiliations:** 1 Health Economics Research Group, Brunel University, Uxbridge UB8 3PH, UK

## Abstract

**Background:**

Peer-reviewed journals are seen as a major vehicle in the transmission of research findings to clinicians. Perspectives on the importance of individual journals vary and the use of impact factors to assess research is criticised. Other surveys of clinicians suggest a few key journals within a specialty, and sub-specialties, are widely read. Journals with high impact factors are not always widely read or perceived as important. In order to determine whether UK surgeons consider peer-reviewed journals to be important information sources and which journals they read and consider important to inform their clinical practice, we conducted a postal questionnaire survey and then compared the findings with those from a survey of US surgeons.

**Methods:**

A questionnaire survey sent to 2,660 UK surgeons asked which information sources they considered to be important and which peer-reviewed journals they read, and perceived as important, to inform their clinical practice. Comparisons were made with numbers of UK NHS-funded surgery publications, journal impact factors and other similar surveys.

**Results:**

Peer-reviewed journals were considered to be the second most important information source for UK surgeons. A mode of four journals read was found with academics reading more than non-academics. Two journals, the *BMJ *and the *Annals of the Royal College of Surgeons of England*, are prominent across all sub-specialties and others within sub-specialties. The *British Journal of Surgery *plays a key role within three sub-specialties. UK journals are generally preferred and readership patterns are influenced by membership journals. Some of the journals viewed by surgeons as being most important, for example the *Annals of the Royal College of Surgeons of England*, do not have high impact factors.

**Conclusion:**

Combining the findings from this study with comparable studies highlights the importance of national journals and of membership journals. Our study also illustrates the complexity of the link between the impact factors of journals and the importance of the journals to clinicians. This analysis potentially provides an additional basis on which to assess the role of different journals, and the published output from research.

## Background

The increasing importance of evidence-based practice in medicine highlights the desirability of clinical practitioners keeping in touch with clinical research. The growing volume of information is available in many different forms, but Schein et al[1] found that the traditional, peer-reviewed journals were the information source considered most important by American surgeons. Many groups have an interest in knowing about the perceived importance and readership of the various journals, including researchers, the users of research and those who determine how researchers should best be assessed as well as those involved in journal publication [2-5].

The issue is complicated by the increasing availability of journals via the internet and also by the sheer number of journals available, for example there were 139 journals in the 2001 'Surgery' category of the Science Citation Index (SCI, from Thomson Scientific, 2004) and many other journals also include papers on surgery.

The introduction of the journal impact factor by the Institute for Scientific Information (ISI, now Thomson Scientific) provided a quantitative shorthand method to assess scholarly journals that was of particular interest to researchers considering where to submit their articles. There is, though, criticism when the use of journal impact factors is extended to cover the assessment of the output of researchers [6-8]. One concern is that different fields have different average journal impact factors. For example clinical specialties, such as surgery, tend to have lower scores than other more basic fields and risk losing out. Research assessments can affect the publication behaviour of authors [9-12] and hence, the more important impact factors are perceived to be, the greater the pressures on researchers to submit papers to journals with high impact factors. At the same time there are attempts to broaden the scope of research assessment by, for example, considering the impact of research on clinical practice[2,13,14].

It is relevant, therefore, to explore the extent of journal readership by clinicians and, as the term 'read' can have many interpretations, to identify the individual journals they rank important for informing their clinical practice. This can only effectively be examined at the level of specific specialties and sub-specialties, though similar patterns may emerge from different specialties. The survey by Schein et al [1] of the reading habits of American surgeons is one of the few studies of these issues in any discipline although there was also a survey of general surgeons passing the UK Intercollegiate Board exam in 1997 [15]. In this context we attempted to provide a comprehensive analysis of the journals read and perceived as important by UK clinicians in a series of specialties, starting with psychiatry [16] and continuing here with surgery.

We conducted a questionnaire survey to identify the relative importance of journals as an information source to UK surgeons, the individual journals of importance to them, further to explore the issues emerging from the previous surveys and the potential implications of these for the assessment of clinical research.

## Methods

Ethics approval was not required for this study as the survey was conducted anonymously using a list of names and addresses taken from the Medical Directory (see below) which is available in the public domain. Prior to the release of the Medical Directory, those listed are given the opportunity to exclude their names from external surveys. All researchers involved are independent of the funders of the project.

### Questionnaire construction

Using the methods we have described elsewhere [17] we constructed a questionnaire containing a list of 39 journals including general medical, specialty and sub-specialty journals either if they contained a large number of NHS-funded surgery papers or if they had a high impact factor relative to other similar journals[18] (See Table 1). Thus the list was derived from two sources:

**Table 1:** Journal names as presented to surgeons in the questionnaire.

Please tick up to ten journals in total that you read or consult on a regular basis *to inform your clinical practice*. If necessary add any that are not listed. From those you have ticked please rank the top three journals (i.e. 1,2 or 3).	
**Journal**	**Tick up to 10 Rank top 3**
Acta Orthopaedica Scandinavica	
Acta Oto-Laryngologica	
American Journal of Surgical Pathology	
American Journal of Transplantation	
Annals of Surgery	
Annals of Surgical Oncology	
Annals of the Royal College of Surgeons of England	
Annals of Thoracic Surgery	
Archives of Surgery	
British Journal of Oral & Maxillofacial Surgery	
British Journal of Plastic Surgery	
British Journal of Surgery	
British Journal of Urology	
BMJ	
Clinical Orthopaedics and Related Research	
Clinical Otolaryngology	
European Journal of Pediatric Surgery	
International Journal of Oral & Maxillofacial Surgery	
Journal of Bone & Joint Surgery – British Volume	
Journal of Endovascular Therapy	
Journal of Internal Medicine	
Journal of Neurology, Neurosurgery & Psychiatry	
Journal of Neurosurgery	
Journal of Pediatric Surgery	
Journal of the American College of Surgeons	
Journal of the Royal Society of Medicine	
Journal of Thoracic and Cardiovascular Surgery	
Journal of Urology	
Journal of Vascular Surgery	
Lancet	
Lasers in Surgery and Medicine	
Liver Transplantation	
Neurosurgery	
Obesity Neurosurgery	
Shock	
Surgery	
Transplant International	
Transplantation	
Transplantation Proceedings	

• *The National Health Service (NHS) Research Outputs Database (ROD) *– The ROD was constructed by the Wellcome Trust[19] and then maintained by the Centre for Information Behaviour and the Evaluation of Research (ciber), City University. It covers the full range of UK biomedical research publications, including basic and clinical sciences, in the peer-reviewed journals contained in the Science and the Social Science Citation Indices from Thomson Scientific. It also includes details of funding acknowledgements. NHS ROD is a subset of ROD which contains details of publications from England with evidence of some element of NHS financial support[20]. A ROD surgery filter, constructed at ciber, was used to extract a list of the leading journals covering 70% of surgery publications on the NHS ROD over the period 1990–1999.

• *Journal Citation Reports 2002 – The Journal Citation Reports^® ^on the Web (JCR^® ^Web) *is a resource from Thomson Scientific for journal evaluation which 'covers more than 7,500 of the world's most highly cited, peer-reviewed journals'. Coverage is both multidisciplinary and international[18]. From the 2002 version we combined the top 20 journals from the specialty of surgery and the UK published journals in the top 20 from the general medical category, both rankings by impact factor.

Surgeons' names and addresses were taken from the Medical Directory 2003/4 CD-ROM (produced by Informa Healthcare, UK in association with the Royal Society of Medicine, London). In this database the names and addresses of surgeons are held under the sub-specialties of surgery which has resulted in some duplication where surgeons are entered as specialising in more than one sub-specialty. Hence 4,400 names were reduced to 2,660 after removal of duplicates and in line with the privacy policy. In addition the sub-specialty divisions were not always the same as the sub-specialty classification described by the Royal College of Surgeons of England and therefore we considered it to be more accurate and informative to ask the surgeons in the questionnaire for details of their sub-specialty/sub-specialties. All consultant surgeons listed with full registration and not retired were included in the questionnaire. We asked the questionnaire recipients to tick up to 10 journals in total that they read or consulted on a regular basis to inform their clinical practice (see Table 1) and invited them to add and tick any that were not listed and from those ticked to rank the top three journals. We also asked them to rank various information sources, including peer-reviewed journals, surgical colleagues and professional meetings and conferences for their role in informing their clinical practice. Responses to the survey were collected over a four-month period early in 2004. The survey was carried out anonymously with no means of identification included on the questionnaires and no reminders were distributed.

### Questionnaire analysis

We transferred the data, using a double-entry procedure to ensure its integrity, from the returned questionnaires into a database for analysis. To verify the names of journals added to the questionnaires we consulted Ulrich's International Periodicals Directory[21] and the internet.

We collated and tabulated the surgeons' responses according to their sub-specialty and academic responsibility and examined the importance of journals as an information source and the relationships between readership, journal rankings, impact factors and numbers of publications. The journal impact factor is 'a measure of the frequency with which the "average article" in a journal has been cited in a particular year or period.'[22] Journal impact factors were obtained from the 2001 edition as the most relevant for analysis of data taken from the NHS ROD 1990–1999 and for numbers of publications an updated, more accurate version of the ROD surgery filter was used.

## Results

2,660 questionnaires were distributed and a total of 1,003 questionnaires were completed and returned (a 38% response rate). Due to some surgeons specialising in more than one discipline the total number of responses to the question concerning sub-specialty (Q4 on questionnaire) was 1,046 (104%). Those surgeons with some academic responsibilities formed 29% of respondents.

A substantial number of surgeons added more journals to the list that had been included in the original questionnaire. The respondents ticked the 39 journals originally listed 4,336 times and the 224 added journal names on 1,316 occasions. Those included in the original questionnaire appear in italics in Tables 2 and 3.

**Table 2:** Percentage surgeons reading selected journals with regard to their clinical work (all journals read by at least 10% of surgeons in one or more of the listed categories).

	**Sub-specialties %**								
					
		**Largest**							
						
**Journals read**	**All**	**General surgery**	**Otolaryng ology**	**Urology**	**Vascular surgery**	**NHS (England) surgery publications 1990–99**	**JIF 2001**	**24 listed journals ranked by JIF**	**139 surgery journals from SCI's ranking by JIF**
*BMJ (UK)*	77.9	81.1	77.8	80.5	87.3	263	6.6	3	-
***Annals of the Royal College of Surgeons of England (UK)***	**61.0**	**77.1**	**48.9**	**48.0**	**72.5**	**838**	**0.5**	**21**	**103**
*British Journal of Surgery (UK)*	48.0	97.2	1.5	14.6	93.1	1510	3.5	4	5
***Lancet (UK)***	**30.8**	**48.0**	**11.9**	**23.6**	**49.0**	**211**	**13.3**	**1**	**-**
*Annals of Surgery*	24.7	47.2	5.9	10.6	29.4	18	6.7	2	1
***Journal of the Royal Society of Medicine (UK)***	**18.6**	**21.2**	**29.6**	**15.4**	**14.7**	**308**	**0.7**	**19**	**-**
*Surgery*	16.7	31.6	3.7	7.3	23.5	37	2.6	8	14
***BJU International (British Journal of Urology) (UK)***	**14.6**	**0.0**	**0.0**	**98.4**	**2.0**	**458**	**1.4**	**12**	**-**
*Clinical Otolaryngology (UK)*	14.3	0.0	97.0	0.0	0.0	123	0.7	19	-
***Journal of Urology***	**12.3**	**1.1**	**0.0**	**87.0**	**0.0**	**54**	**3.2**	**5**	**-**
*British Journal of Plastic Surgery (UK)*	10.7	1.7	3.7	0.0	0.0	372	0.8	17	67
***Journal of Vascular Surgery***	**8.3**	**9.3**	**0.0**	**0.8**	**76.5**	**76**	**3.1**	**6**	**7**
*Archives of Surgery*	8.1	18.6	0.7	0.0	10.8	19	2.8	7	11
**Journal of Laryngology and Otology (UK)**	**8.0**	**0.0**	**58.5**	**0.0**	**0.0**	**323**	**0.5**	**21**	**-**
*Journal of the American College of Surgeons*	7.2	15.8	1.5	0.8	4.9	17	2.4	9	16
***Acta Oto-Laryngologica***	**6.3**	**0.0**	**42.2**	**0.0**	**0.0**	**80**	**0.8**	**17**	**-**
European Journal of Vascular and Endovascular Surgery (UK)	6.1	8.2	0.0	0.0	56.9	0	1.5	11	40
**European Journal of Surgical Oncology (UK)**	**5.0**	**12.7**	**0.0**	**0.0**	**2.0**	**2**	**1.3**	**14**	**45**
Colorectal Disease (UK)	4.6	11.9	0.0	0.0	0.0	N/A	N/A	N/A	-
**Laryngoscope**	**4.0**	**0.0**	**28.1**	**0.0**	**0.0**	**18**	**1.4**	**12**	**-**
Archives of Otolaryngology – Head and Neck Surgery	2.2	0.0	15.6	0.0	0.0	25	1.1	16	52
***Journal of Endovascular Therapy (Journal of Endovascular Surgery)***	**2.2**	**1.7**	**0.0**	**0.0**	**20.6**	**8**	**2.1**	**10**	**26**
Otology & Neurotology (American Journal of Otology)	1.4	0.0	10.4	0.0	0.0	56	1.2	15	-
**Phlebology (UK)**	**1.4**	**2.8**	**0.0**	**0.0**	**12.7**	**64**	**0.5**	**21**	**114**

Journal titles in italics were included in the original questionnaire; SCI, Science Citation Index from Thomson Scientific; -, Journals not included in Thomson Scientific's surgery rankings in the SCI; N/A, Journal without a JIF

**Table 3:** Percentage surgeons ranking selected journals 1, 2 or 3 in importance with regard to their clinical work (all journals ranked by at least 20% of surgeons in one or more sub-specialty).

	**Sub-specialties %**										
	
**Journals ranked**	**All**	**Cardio thoracic surgery**	**General surgery**	**Neuro surgery**	**Oral & Maxillo facial surgery**	**Otolaryngology**	**Paediatric surgery**	**Plastic surgery**	**Trauma & Ortho paedic surgery**	**Urology**	**Vascular surgery**
*British Journal of Surgery (UK)*	40	2	91	0	0	0	48	4	3	5	79
***BMJ (UK)***	**36**	**39**	**37**	**15**	**38**	**44**	**29**	**30**	**17**	**50**	**32**
*Annals of the Royal College of Surgeons of England (UK)*	24	11	38	13	15	22	27	28	13	15	26
***BJU International (British Journal of Urology) (UK)***	**13**	**0**	**2**	**0**	**0**	**0**	**15**	**0**	**1**	**94**	**1**
*Clinical Otolaryngology (UK)*	13	0	0	3	6	90	10	4	0	0	0
***Journal of Urology***	**10**	**0**	**1**	**0**	**0**	**0**	**8**	**0**	**0**	**81**	**0**
*British Journal of Plastic Surgery (UK)*	7	0	0	0	15	2	2	82	0	0	0
**Journal of Laryngology and Otology (UK)**	**6**	**0**	**0**	**0**	**0**	**47**	**2**	**3**	**0**	**0**	**0**
*Journal of Bone & Joint Surgery – British Volume (UK)*	6	0	0	3	0	0	0	0	87	0	1
***British Journal of Oral & Maxillofacial Surgery (UK)***	**5**	**0**	**0**	**0**	**89**	**0**	**0**	**7**	**0**	**0**	**0**
European Journal of Vascular and Endovascular Surgery (UK)	5	0	7	0	0	0	2	0	0	0	51
***Journal of Vascular Surgery***	**5**	**0**	**6**	**0**	**0**	**0**	**2**	**0**	**0**	**0**	**49**
*Journal of Thoracic and Cardiovascular Surgery*	4	96	0	0	0	0	4	0	1	0	0
***Annals of Thoracic Surgery***	**4**	**89**	**0**	**0**	**0**	**0**	**4**	**0**	**0**	**0**	**0**
Plastic and Reconstructive Surgery	4	0	0	0	11	0	0	48	0	0	0
***Journal of Neurosurgery***	**3**	**0**	**0**	**83**	**0**	**0**	**4**	**0**	**0**	**0**	**0**
*Neurosurgery*	3	0	0	63	0	1	2	0	0	0	0
***International Journal of Oral & Maxillofacial Surgery (UK)***	**3**	**0**	**0**	**0**	**55**	**0**	**0**	**0**	**0**	**0**	**0**
*Journal of Pediatric Surgery*	3	0	0	0	0	0	44	0	0	2	0
**Journal of Hand Surgery (British and European) (UK)**	**3**	**0**	**0**	**3**	**0**	**0**	**0**	**20**	**14**	**0**	**0**
Journal of Bone and Joint Surgery (American)	3	0	0	0	0	0	0	0	37	0	0
**European Journal of Cardiothoracic Surgery**	**2**	**30**	**0**	**0**	**0**	**0**	**4**	**0**	**0**	**0**	**0**
British Journal of Neurosurgery (UK)	2	0	0	40	0	0	2	0	0	0	0
***Clinical Orthopaedics and Related Research***	**2**	**0**	**0**	**0**	**0**	**1**	**0**	**0**	**23**	**0**	**0**
*Journal of Neurology, Neurosurgery & Psychiatry (UK)*	1	0	0	28	0	0	0	0	0	0	0
***European Journal of Pediatric Surgery***	**1**	**0**	**0**	**0**	**0**	**1**	**21**	**0**	**0**	**0**	**0**

Journal titles in italics were included in the original questionnaire.

Peer-reviewed journals were considered important by 72% of surgeons and were the second highest information source after professional meetings and conferences at 92%, the third highest being surgical colleagues at 64%. All three of these information sources were consulted by at least 95% of respondent surgeons. Internet sources were considered important by 10% of respondents but were used by 64%.

**Figure 1:**
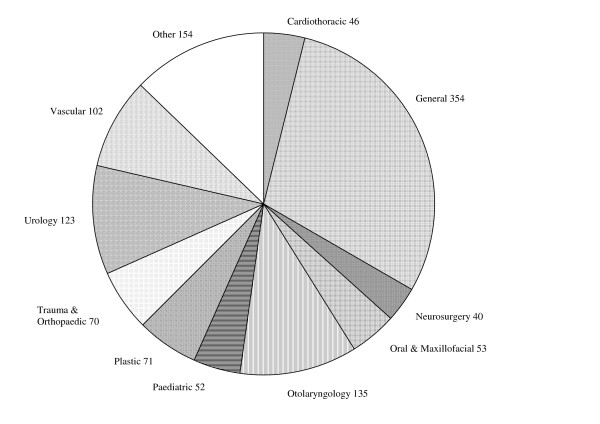
Number of respondents within each surgical sub-specialty.

**Figure 2:**
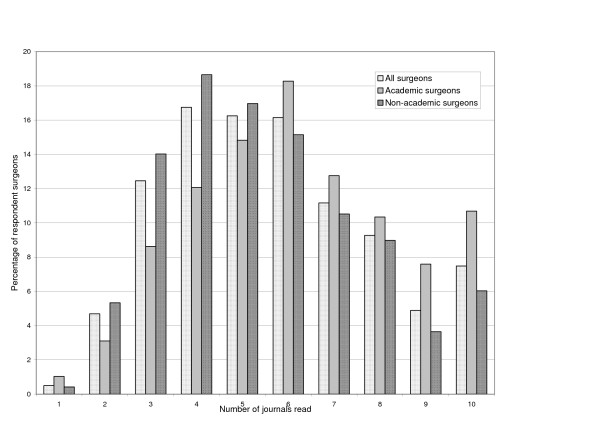
The number of journals read by respondent surgeons.

**Figure 3:**
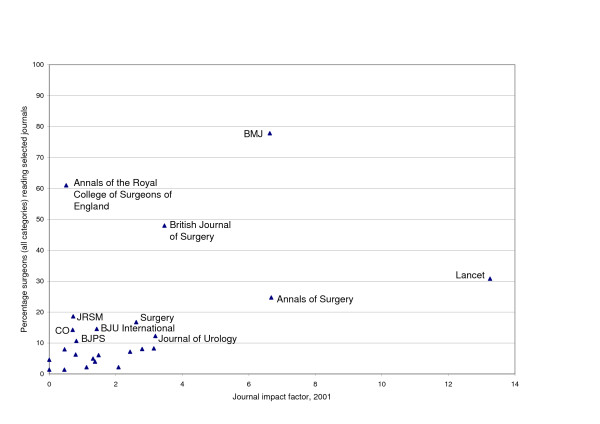
Percentage respondent surgeons reading selected journals v. journal impact factor 2001 (all journals listed in Table 1). Abbreviations : BJPS: British Journal of Plastic Surgery CO: Clinical Otolaryngology JRSM: Journal of the Royal Society of Medicine

Further results of the survey are presented in Tables 2 and 3 and illustrated in Figures 1, 2, 3. The modal number of journals read by respondent surgeons was 4 though for those with academic responsibilities this rose to 6 (Figure 2). A statistically significant difference was found between the number of journals read by academics and non-academics (t = -2.90, p = 0.010). There was some variation in readership across sub-specialties with urologists reading the least (mode 3, with 35% reading 3 or less journals and 3% reading 10+ journals) and oral and maxillofacial surgeons reading the most (bimodal 4 and 6, 9% reading 3 or less journals and 13% reading 10+ journals).

In Table 2 we have presented the data on journal impact factors in a number of ways including (in the last column) their position in the ISI ranking of 139 surgery journals, which does not include either general medical journals or some sub-specialty journals which appear in different rankings.

## Discussion

As found by Schein et al, journals were considered to be one of the most important information sources to inform the clinical practice of surgeons. Nevertheless, with mode of 4 for journals read by surgeons overall, and with 20% of 'non-academic' surgeons reading three journals or less, many surgeons are exposed to just a few journals.

The data in Tables 2 and 3 address the issue of a few key journals and show the same three journals being read, and rated most highly, by substantially more surgeons than any other journals. The three are: *BMJ*, a general medical journal, the *Annals of the Royal College of Surgeons of England*, a specialty journal, and *British Journal of Surgery *which traditionally has published papers in breast, upper and lower GI, vascular, endocrine and surgical sciences. The *British Journal of Surgery *is ranked by the greatest number of surgeons overall but, as with its readership, this is based on its importance for three sub-specialties: general surgery (the largest number of respondents), paediatric surgery and vascular surgery. The *BMJ *and the *Annals of the Royal College of Surgeons of England *are ranked as important by surgeons in all sub-specialties, though to varying degrees. Our findings are broadly in line with the small survey of 1997 candidates for the general surgery examination which showed that they read the *British Journal of Surgery *most widely, followed by the *BMJ*[15]. This suggests that at least across the board there are just a few key journals; and beyond the three above, the only journals of importance to more than 20% in any of the nine surgery sub-specialties are sub-specialty journals. Within each sub-specialty between three and five individual journals are considered important by more than 20% of surgeons. This gives a total of 26 journals that are important to at least 20% of any one sub-specialty, a figure which represents a small proportion of the total number of journals read by surgeons.

Consideration of the nationality of the most widely read and highly rated journals introduces a further dimension into the analysis. UK based journals feature prominently amongst those general medical and specialty journals most widely read by surgeons overall (Table 2) but at sub-specialty level, for journals ranked as important, the picture is more varied (Table 3). For example, in the two sub-specialties with least respondents (neurosurgery and cardiothoracic surgery), the journals read most widely are not UK based and yet they are read by at least 80% of surgeons within their respective sub-specialties. These findings of bias, above sub-specialty level, towards local journals are consistent with Schein et al's[1] survey of American surgeons where the top three general medical journals, and the top five surgical journals, were all found to be American. The *British Journal of Surgery *was rated as one of the three most popular journals by only 0.5% of American surgeons. Yet Schein et al also reported the results of an international e-mail audit of general surgeons where 33% of respondents considered the *British Journal of Surgery *the 'best' general surgical journal in the world – a higher figure than for any other journal [1], even the Annals of Surgery which has the highest impact factor for any surgical journal and is rated highest by the most US surgeons. In the Netherlands, national journals were similarly reported to be very important for the dissemination of research findings to clinicians[2]. The apparent importance of national journals to surgeons is interesting in light of the increasing availability of journals and bibliographic databases over the internet and also Tompkins et al's findings that the proportion of nationally produced papers published in the highest rated British and American journals had decreased over the period 1983 to 1998 as the journals became more international[23]. In US journals this decrease was from 87.5% to 68.8% and in the one British journal included in the analysis (*British Journal of Surgery*) from 74.8% to 47.1%. Tompkins et al found the sources of the greatest increases in article numbers were European and Asian authors. Nationality of publications had previously been found to play an important part in the flow of information from research to clinical practice via UK clinical guidelines[24].

There are many professional organisations within the UK for surgeons, either generally or within the sub-specialties, and many of these organisations produce or support specific journals. Similarly, many journals are supported by professional organisations based in the USA. These journals are usually available to members of the organisations either as part of their membership or at a significantly reduced rate. The percentage of surgeons likely to subscribe to a journal and the level of rate reduction applied to the journal result in a complicated picture overall with regard to readership. Membership journals could perhaps, be expected to have relatively high readership levels but not necessarily high rankings of importance. The *Annals of the Royal College of Surgeons of England*, a membership journal for the Royal College, illustrates this with very high readership levels but more modest numbers of surgeons ranking it as important. *British Journal of Surgery *is a membership journal of the Association of Surgeons of Great Britain and Ireland, the professional association for general surgeons, and is perhaps more of a sub-specialty journal for the largest sub-specialty of general surgery rather than a specialty surgery journal. It has a relatively high readership level overall, though not the highest, and is considered the most important journal by the largest number of surgeons. The issue of membership journals adds a further level to the analysis by nationality.

The final issue is a comparison of the journals read most widely by surgeons and journal impact factor (Figure 3). Here a complex picture emerges with no clear consistent relationship as was found previously.

The issue of journal impact factor in this survey is complicated by several considerations including the apparent preference of UK surgeons for UK journals, which generally have lower impact factors than US journals, and the issue of membership journals. Looking just at the journals included in the ISI surgery list from Thomson Scientific, only three of the top ten appear among the 24 journals listed on Table 2 as having the highest readership by UK surgeons. Indeed the *Annals of the Royal College of Surgeons of England*, is second on the list for readership (and third on the list for importance to clinical practice – see Table 3) but in position 103 out of 139 when ranked by impact factor. When the general medical journals are brought into the analysis, however, a rather different picture emerges because, as noted, two of the most read journals are the *BMJ *and the *Lancet *and they have relatively high impact factors. Therefore, apart from the *Annals of the Royal College of Surgeons of England*, the five most read journals have the highest impact factors of all 24 journals in Table 2. With its many different facets, the complex relationship between readership by clinicians and impact factors confirms the need for caution in their use in the assessment of outputs from individual researchers.

Including a list of journals in the questionnaire automatically introduces bias against those not listed. Although attempts were made to reduce this to a minimum by using precise inclusion criteria some element of bias is to be expected and the names of the journals that were included in the questionnaire have been identified in the tables to allow consideration to be made. The response rate to our survey of 38% is comparable to Schein's analysis of American surgeon's with 37% of questionnaires suitable for analysis. This response level suggests caution should be exercised in extrapolating the findings to the whole body of UK surgeons as the opinions of non-respondents may differ from those of the respondents. Given the anonymity of the survey, our knowledge about the representativeness of the responders is limited with regard to, for example, geographical distribution of respondents across the UK, response rates within each of the sub-specialties, age and sex of respondents. Furthermore, we recognise that the situation is liable to change particularly as electronic access to journals becomes more widespread and this survey is cross-sectional and therefore unable to track changes in readership levels or journal impact factors over time. Nevertheless, overall, the findings presented here related to each of the issues provide a firmer evidence base than previously existed and should help inform the decisions made by researchers and those who assess them, and by the readers and editors of journals. They can be used to address the issues raised earlier.

Overall, the evidence potentially provides an additional basis on which to assess the role of different journals and the published output from research. Furthermore, the importance of the nationality of journals to clinicians suggests the type of survey being reported here could usefully be extended to other countries.

## Conclusion

UK surgeons consider peer-reviewed journals to be an important information source. The mode for journals read is four with academics reading more than non-academics. For UK surgeons a few journals are key across all sub-specialties and others within sub-specialties. UK journals are generally preferred by UK surgeons and readership patterns are influenced by membership journals. Some key journals do not have high impact factors.

## Competing interests

The author(s) declare that they have no competing interests.

## Authors' contributions

MB and SH prepared grant submissions for the project, TJ, MB and SH were involved in the planning, preparation and approval of the original questionnaire. TJ conducted the questionnaire survey and data collection. TJ carried out the data analysis with additional intellectual input from MB and SH. All authors read and approved the final manuscript.

## Pre-publication history

The pre-publication history for this paper can be accessed here:



## References

[B1] Schein M, Paladugu R, Sutija V, Wise L (2000). What American surgeons read: A survey of a thousand Fellows of the American College of Surgeons. Curr Surg.

[B2] Royal Netherlands Academy of Arts and Sciences The Societal Impact of Applied Health Research. Towards a Quality Assessment System. http://www.knaw.nl/publicaties/pdf/20021098.pdf.

[B3] Lewison G (2002). Researchers' and users' perceptions of the relative standing of biomedical papers in different journals. Scientometrics.

[B4] Traynor M, Rafferty A (2001). Bibliometrics and a culture of measurement. J Adv Nurs.

[B5] Blecic D D (1999). Measurements of journal use: an analysis of the correlations between three methods. Bull Med Libr Assoc.

[B6] Seglen PO (1998). Citation rates and journal impact factors are not suitable for evaluation of research. Acta Orthop Scand.

[B7] Abbasi K (2004). Let's dump impact factors. BMJ.

[B8] Frank M (2003). Impact factor: arbiter of excellence?. J Med Libr Assoc.

[B9] Croxson B, Hanney S, Buxton M (2001). Routine monitoring of performance: what makes health research and development different?. J Health Serv Res Policy.

[B10] Butler L (2003). Explaining Australia's increased share of ISI publications; the effects of a funding formula based on publication counts. Res Policy.

[B11] Walford L (1999). The Research Assessment Exercise: its effect on scholarly journal publishing. Learn Publ.

[B12] Cave M, Hanney S, Henkel M, Kogan M (1997). The Use of Performance Indicators in Higher Education: The Challenge of the Quality Movement.

[B13] Buxton M, Hanney S (1996). How can payback from health services research be assessed?. J Health Serv Res Policy.

[B14] Hanney SR, Grant J, Wooding S, Buxton MJ (2004). Proposed methods for reviewing the outcomes of health research: the impact of funding by the UK's 'Arthritis Research Campaign'. Health Res Policy Syst.

[B15] Macleod DAD (1998). BMJ is second most popular journal among surgeons. BMJ.

[B16] Jones T, Hanney S, Buxton M, Burns T (2004). What British psychiatrists read. Questionnaire survey of journal usage among clinicians. Br J Psychiatry.

[B17] Jones T, Hanney S, Buxton M, Rippon I (2005). Journals used for the publication of English psychiatry, surgery and paediatrics research. Aslib Proc: New Information Perspectives.

[B18] Thomson Scientific, Philadelphia ISI Journal Citation Reports: JCR Glossary. http://jcrweb.com/www/help/hjcrgls2.htm.

[B19] Dawson G, Lucocq B, Cottrell R, Lewison G (1998). Mapping the Landscape: National Biomedical Research Outputs 1988-95.

[B20] Wellcome Trust, NHS Executive (2001). Putting NHS research on the map. An analysis of scientific publications in England, 1990-97.

[B21] Bowker R (2003). Ulrich's international periodicals directory: including irregular serials and annuals.

[B22] Garfield E The ISI impact factor. http://scientific.thomson.com/free/essays/journalcitationreports/impactfactor/.

[B23] Tompkins RK, Yo CY, Donovan AJ (2001). Internationalization of general surgical journals - Origin and content of articles published in North America and Great Britain from 1983 to 1998. Arch Surg.

[B24] Grant J, Cottrell R, Cluzeau F, Fawcett G (2000). Evaluating "payback" on biomedical research from papers cited in clinical guidelines: applied bibliometric study.. BMJ.

